# Laboratory Assessment of Factor VIII Inhibitors: When Is It Required? A Perspective Informed by Local Practice

**DOI:** 10.3390/jcm14010013

**Published:** 2024-12-24

**Authors:** Emmanuel J. Favaloro, Jennifer Curnow, Leonardo Pasalic

**Affiliations:** 1 Haematology, Sydney Centres for Thrombosis and Haemostasis, Institute of Clinical Pathology and Medical Research (ICPMR), NSW Health Pathology, Westmead Hospital, Westmead, NSW 2145, Australia; leonardo.pasalic@health.nsw.gov.au; 2 School of Dentistry and Medical Sciences, Faculty of Science and Health, Charles Sturt University, Wagga, NSW 2650, Australia; 3 School of Medical Sciences, Faculty of Medicine and Health, University of Sydney, Westmead Hospital, Westmead, NSW 2145, Australia; jennifer.curnow@sydney.edu.au; 4 Clinical Haematology, Sydney Centres for Thrombosis and Haemostasis, Westmead Hospital, Westmead, NSW 2145, Australia; 5 Westmead Clinical School, University of Sydney, Westmead, NSW 2145, Australia

**Keywords:** factor VIII, inhibitors, congenital hemophilia A, acquired hemophilia A, Bethesda assay

## Abstract

This perspective discusses the critical role of laboratory assessments in assessing factor VIII (FVIII) inhibitors. These are auto- and alloantibodies that can develop against both endogenous and exogenous FVIII, respectively. Assessment for inhibitors represents a key part of the management of both congenital hemophilia A (CHA), an inherited deficiency, and acquired hemophilia A (AHA), an autoimmune condition. Both conditions pose significant bleeding risks, necessitating careful monitoring of FVIII levels and inhibitor presence and level. Laboratory assays, particularly the Bethesda assay, are essential for detecting these inhibitors and assessing their levels. The complexities of FVIII inhibitor kinetics may pose challenges to interpretation of assay results, such that even normal FVIII levels do not always exclude inhibitor presence. Clinical practice guidelines recommend ongoing monitoring of AHA/CHA patients until inhibitors are no longer detectable. Overall, timely laboratory evaluations are essential to optimizing treatment strategies for patients with hemophilia, aiming to improve patient outcomes and quality of life. We summarize our approach to the laboratory assessment of FVIII inhibitors, as reflecting our perspective and as informed by local practice.

## 1. Introduction

Factor VIII (FVIII) inhibitors are antibodies that develop against FVIII, either endogenous FVIII (as in the case of acquired hemophilia A (AHA)) or exogenous FVIII (as in the case of congenital hemophilia A (CHA)) [1,2,3,4,5]. In turn, CHA represents an inherited deficiency of FVIII, whereas AHA represents an acquired deficiency of FVIII, typically caused by FVIII inhibitors. FVIII is an important procoagulant protein that facilitates secondary hemostasis or clot formation [6]. Thus, deficiency in FVIII, either inherited (i.e., CHA) or acquired (i.e., AHA), represents a bleeding risk, with this risk in part dependent on the level of plasma FVIII present in any individual [7]. Hemophilia is defined as severe when FVIII levels are <1% of normal (or <1 U/dL), moderate when FVIII levels are 1 to <5% (or U/dL), and mild when FVIII levels are 5 to 40% (or U/dL). Hemophilia A is a sex-linked disorder, considered one of the most common inherited bleeding disorders, affecting around one in five thousand males, or around one in ten thousand people [7]. Because of bleeding risk, people with CHA often need to be treated to reduce their risk of bleeding, perhaps for elective surgery, or in some cases managed for active bleeding that has occurred spontaneously or because of some trauma. Indeed, people with severe CHA may be under prophylactic treatment, since such severely affected people may otherwise suffer regular spontaneous episodes of bleeding, which may lead to a reduced quality of life, especially if frequent bleeds occur within joints [8].

In the past, treatment for CHA mostly consisted of replacement FVIII therapy, initially with plasma-derived FVIII (pdFVIII), then recombinant FVIII (rFVIII), and subsequently extended half-life rFVIII (EHL-rFVIII) [9,10,11,12,13]. There is now a wide armamentarium of possible therapies, such as bypassing agents, FVIII mimetics or hemostasis rebalancing agents, porcine FVIII, and even gene therapy [9,10,11,12,13]. The FVIII mimetic emicizumab is now very widely used, but bleeds still occur with trauma, with surgery, and occasionally spontaneously, so replacement FVIII concentrates still represent a common treatment modality [14,15,16]. Inhibitors may develop in CHA at any time during treatment with replacement FVIII therapy, whether pdFVIII, rFVIII, or EHL-rFVIII. Indeed, inhibitors may occur in up to 25% of such treated individuals. Instead, inhibitors that develop in AHA develop against endogenous FVIII and represent an acquired autoimmune disorder. AHA is associated with increasing age, pregnancy, infection, autoimmune disorders and malignancies (including monoclonal gammopathy of undetermined significance [MGUS]), or certain medications [17,18].

FVIII inhibitors act against the FVIII protein to reduce the quantity (e.g., through increased clearance) or to inhibit its function. Thus, FVIII inhibitors lead to reduced levels of functional FVIII and thus are also risk factors for bleeding. Moreover, FVIII inhibitors may render FVIII replacement therapy ineffective, depending on the level of inhibitor.

The hemostasis or coagulation laboratory may be involved at several stages of clinical investigation, for example for assessment of FVIII levels, for measurement of FVIII inhibitors, either in CHA during treatment and therapy monitoring, or in suspected AHA [2,19]. In this perspective article, we evaluate at which stages the laboratory may be called upon to measure FVIII inhibitors. In essence, we describe our local experience, in part guided by international guidelines [3,4,5,20,21] and also local Australian guidelines [22]. In addition, local guidance is summarized in Table 1.

## 2. Materials and Methods

This manuscript reflects a perspective article that describes our local practice, as informed by our experience and as guided by international [3,4,5,20,21] and local Australian guidelines [22]. To enable our perspective to develop, we provide data on FVIII inhibitory testing from our laboratory for the past decade. These data were extracted from our laboratory information system (LIS) and covers the years 2015–2024 inclusive. Patients were separately identified as having either congenital or acquired hemophilia.

Additional experience with the inter-relationships of FVIII level, FVIII inhibitor level, and activated partial thromboplastin time (aPTT) is also detailed to further support our perspective.

## 3. Results

### 3.1. The Theoretical Relationship Between aPTT and FVIII Levels

**Figure 1:**
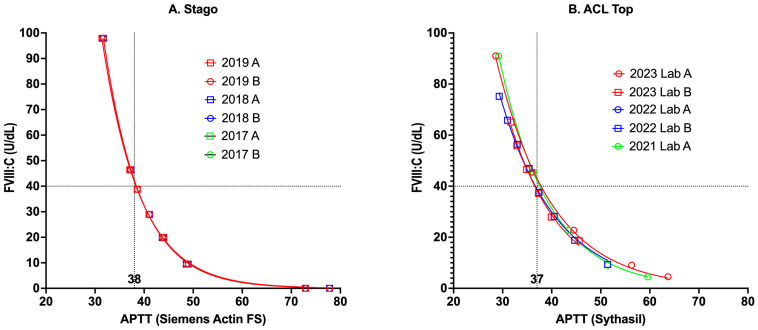
The theoretical relationship between the aPTT and the FVIII level can be highlighted by performing in vitro assessments. Commercial FVIII-deficient plasma (i.e., 0% FVIII) is diluted into normal plasma (usually a pool with theoretical 100% FVIII) to generate a factor-level dose response curve: (**A**) Historical data using a Stago analyzer and evaluating different lots of aPTT reagent (Siemens Actin FS) in three different years and comparing an evaluation reagent (‘A’) with a reference reagent (‘B’). Despite the change in aPTT reagent lots, the response curves were nearly identical, showing excellent comparability between aPTT lots using this method. An aPTT of 38 s (s) represents the normal range cut-off value, meaning an abnormal aPTT if >38 s. (**B**) More recent data using aCL Top analyzers and evaluating different lots of aPTT reagent (Synthasil) in three different years and as performed in two different laboratories (‘A’ and ‘B’). Again, despite the change in aPTT reagent lot and assessment in different laboratories, the response curves were nearly identical, again showing excellent comparability between aPTT lots using this method. An aPTT of 37 s represents the normal range cut-off value, meaning an abnormal aPTT if >37 s. In both situations (i.e., Figure 1A,B), the FVIII sensitivity cutoff was around 40%, meaning levels of FVIII <40% would yield an abnormal aPTT.

Figure 1 provides some laboratory data to highlight the relationship between the aPTT and FVIII levels, as reflective of the test reagents in use in our laboratory in the past (Figure 1A) and currently (Figure 1B). With progressive reduction in FVIII comes increasing prolongation of the aPTT.

### 3.2. The Theoretical Relationship Between FVIII Inhibitor Titer and FVIII Levels

**Figure 2:**
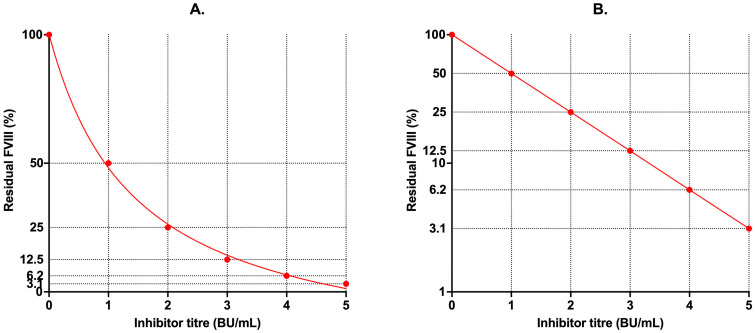
The theoretical relationship between the residual FVIII level (%) and the inhibitor level (Bethesda Units [BU]/mL). If plotted on a linear scale (**A**), the relationship is curvilinear and similar to the relationship between factor level and aPTT (Figure 1). However, the relationship is usually plotted on a log-linear scale (**B**) and usually only to a level of 2 BU/mL, with levels above 2 BU/mL evaluated in serial dilutions of patient plasma.

Figure 2 shows the theoretical relationship between the FVIII inhibitor titer and FVIII levels. In brief, the FVIII inhibitor assay is performed as a modified FVIII assay, where serial dilutions of patient plasma are mixed with normal plasma (in theory containing ~100% FVIII), and FVIII assays are then performed. These FVIII assays in turn are modifications of aPTT assays, where patient plasma is mixed with factor deficient plasma and then an aPTT assay performed.

The relationship, like that of the FVIII level and aPTT (as shown in Figure 1), is also curvilinear (Figure 2A), although it is more common to plot the relationship on a log-linear scale, as shown in Figure 2B.

### 3.3. The Real-World Relationship Between aPTT, FVIII Inhibitor Titre and FVIII Levels

Laboratories can perform assays to assess the presence of FVIII inhibitors, and also assess the level of inhibitor, in either CHA or AHA patients. The classical assay is called the Bethesda assay, named after the location of its ‘inventor’, as potentially modified using the Nijmegen modifications [19].

**Figure 3:**
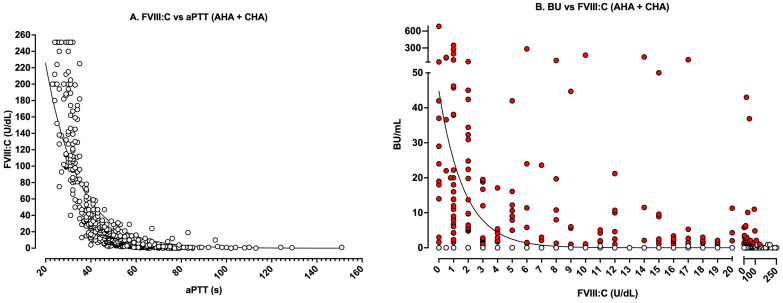
Real-world data showing the relationship between (**A**) FVIII:C and aPTT and (**B**) inhibitor level (in BU/mL) vs. FVIII:C in all patients with acquired (AHA) or congenital (CHA) hemophilia tested in our laboratory from 2015 to 2024 inclusive. In Figure 3B, red dots indicate data with ≥1 BU/mL; clear dots indicate <1 BU/mL or no inhibitor.

Figure 3 shows real-world data from our laboratory over the past decade (2015–2024 inclusive), for all patient samples where an aPTT, FVIII inhibitor titre and FVIII levels were performed and where patients were identified to be either CHA or AHA. These data capture all aPTT data with a corresponding FVIII level (Figure 3A) and all FVIII levels with a corresponding inhibitor level (Figure 3B) for all assessed patients. In both situations, i.e., FVIII:C vs. aPTT (Figure 3A) or inhibitor level vs. FVIII:C (Figure 3B), the data tend to follow a linear-log relationship (non-linear curves in each figure), similar to theoretical findings shown in Figure 1 and Figure 2, although some points do not sit on these curves, especially in Figure 3B.

**Figure 4:**
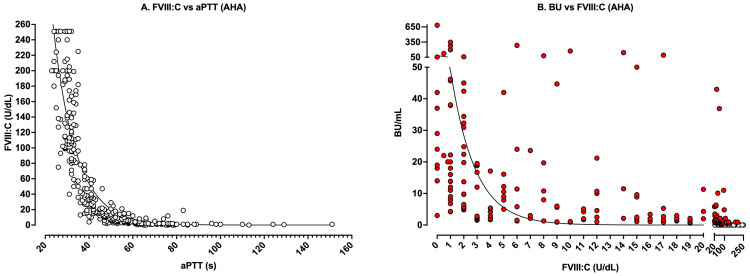
These data capture (**A**) all aPTT data with a corresponding FVIII level and (**B**) all FVIII levels with a corresponding inhibitor level for all AHA patients tested in our laboratory from 2015 to 2024 inclusive. In Figure 4B, red dots indicate data with ≥1 BU/mL; clear dots indicate <1 BU/mL or no inhibitor. Again, the data tend to follow a linear-log relationship (non-linear curves in each figure), although some points (especially in Figure 4B) do not sit on this curve. Note, however, that most data points in Figure 4B comprise inhibitor positive samples.

**Figure 5:**
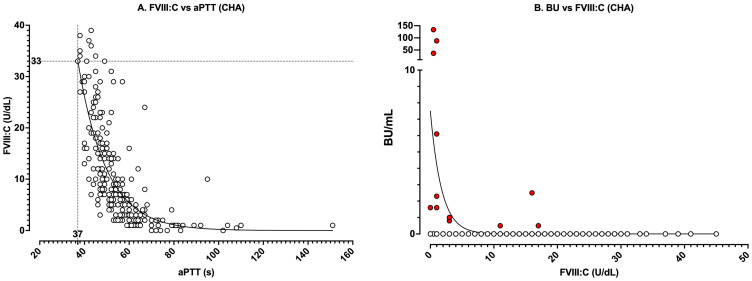
These data capture (**A**) all aPTT data with a corresponding FVIII level and (**B**) all FVIII levels with a corresponding inhibitor level for all CHA patients tested in our laboratory from 2015 to 2024 inclusive. In Figure 5B, red dots indicate data with ≥1 BU/mL; clear dots indicate <1 BU/mL or no inhibitor. Again, the data tend to follow a linear-log relationship (non-linear curves in each figure), although some points (especially in Figure 5B) do not sit on this curve. Note, however, that most data points in Figure 5B comprise inhibitor negative samples.

Figure 4 and Figure 5 show the same data respectively separated into AHA or CHA to highlight similarities and differences in test patterns between the two patient categories. When data are separated into AHA vs. CHA, several distinct patterns emerge. Although the relationship between FVIII:C and aPTT is similar between AHA vs. CHA (i.e., Figure 4A vs. Figure 5A), the limits are not. By definition, CHA represents hereditary deficiency of FVIII:C (i.e., values ≤ 40 U/dL; Figure 5A), with levels identifying clinical bleeding severity.

Also of interest, laboratories can use data from CHA patients to identify the theoretical sensitivity of their aPTT reagent to FVIII deficiency. In Figure 5A, using the log-linear curve, a cutoff of 37 s (being the current upper limit of our normal reference range) yields a value of 33 U/dL of FVIII:C, which is also similar to the value identified in Figure 1 using in vitro dilution studies.

For the FVIII vs. BU relationship, clearer differences between AHA and CHA also become apparent. As noted, most data points in Figure 5B represent negative inhibitor findings, since inhibitors are not identified in most cases of CHA testing. In contrast, for AHA (Figure 4B), one would expect to find inhibitors against FVIII, since this defines AHA. Although the data for both CHA and AHA tend to follow a log-linear curve, there is some variation in the relationship between FVIIIC and BU; however, some patterns emerge. First, in the AHA dataset, FVIII:C levels below 20 U/dL were all associated with inhibitors ≥1 BU/mL (Figure 4B), which of course makes sense given the definition of BU. However, because of complex kinetics, the actual inhibitor titer for any given level of FVIII:C varied widely. Moreover, although levels of FVIII:C above 50 U/dL were rarely associated with inhibitors, the presence of inhibitors could not always be excluded.

## 4. Discussion

### 4.1. Theoretical vs. Real-World Relationships Between aPTT and FVIII Levels

Theoretical and real-world relationships between aPTT, FVIII levels, and FVIII inhibitor titers have some similarities and also several differences. Theoretical relationships (Figure 1 and Figure 2) are inherently cleaner than real-world relationships, since the relationships are more direct and less influenced by additional confounders. That is, in Figure 1, the level of only one specific factor (i.e., FVIII) is altered, generating a clear relationship between FVIII and the aPTT. Similarly, only FVIII inhibitors are considered in the theoretical relationship between FVIII and FVIII inhibitors (Figure 2). In contrast, in real-world patients, the aPTT is not only affected by the level of FVIII but also the level of other coagulation factors (i.e., FIX, FXI, FXII) [23,24], with these often not assessed for investigated patients. Also, in real-world data, patients may present with additional confounders and may, for example, have a lupus anticoagulant. This means that the relationship between aPTT values vs. FVIII levels for real-world patients are not as clearly concordant as in Figure 1.

Similarly, although the relationship between FVIII and aPTT in AHA (Figure 4A) vs. CHA (Figure 5A) patients is broadly similar, the relationship between FVIII and inhibitor titer in AHA (Figure 4B) vs. CHA (Figure 5B) patients is not. This is because FVIII levels in AHA patients inherently return to normal as the FVIII inhibitor disappears, whereas patients with CHA will have low levels of FVIII even when inhibitors are not present.

### 4.2. Use of Theoretical vs. Real-World Data to Evaluate aPTT Sensitivity to Factor Levels

Use of the in vitro serial dilution method to generate a theoretical sensitivity of any aPTT reagent to specific factor sensitivity (e.g., Figure 1 for FVIII) provides a reasonable real-time method that usually compares well with ex vivo methods (Figure 5A), as also noted in several past publications [23,24]. Moreover, the in vitro method can be applied to identify the sensitivity of reagents to other factor deficiencies [23,25,26]. Instead, the ex vivo method requires a large number of accumulated samples, which will prove difficult for other rarer factor deficiencies, and cannot provide real-time sensitivity data, since it takes time to accumulate sufficient samples [23,25,26].

### 4.3. Theoretical vs. Real-World Relationships Between FVIII Levels and FVIII Inhibitor Levels

For inhibitors, one Bethesda unit (BU) of inhibitor is that which inactivates 50% of the residual FVIII activity [19]. Thus, for a CHA patient, 1 BU of FVIII inhibitor will reduce the efficacy of replacement therapy by 50%. At a level of >5 BU, FVIII replacement therapy is considered to be ineffective, and alternate strategies (e.g., bypass therapy) are required to manage bleeding symptoms [4,5,20,21]. Prophylaxis of bleeding can be achieved with alternative therapies such as FVIII mimetics, with emicizumab being the most commonly used agent currently [15,16]. However, when measuring FVIII levels and inhibitor titers in patients taking emicizumab, further assay modifications are required, including the use of bovine components [27,28,29,30,31].

In a patient with AHA with historically normal FVIII levels, 1 BU of FVIII inhibitor will theoretically reduce a historical level of 100% (or U/dL) to 50%. 5 BU of FVIII inhibitor will theoretically reduce a historical level of 100% (or U/dL) to 3% (Figure 2B) or essentially turn a normal FVIII level into one that can be considered equivalent to a moderate CHA [7]. Figure 2 shows the theoretical relationship between FVIII inhibitor level and FVIII level. However, the real-world situation is more complex than this, in part due to the variable kinetics of FVIII inhibitors. FVIII inhibitors are time- and temperature-dependent. The classical BU of FVIII inhibitors indicates a laboratory incubation step of 2 h at 37 °C to stabilize the inhibitor binding to FVIII and thus better represent the in vivo relationship [19]. In AHA in particular, FVIII inhibitors may have ‘second-order kinetic’ profiles, meaning a non-linear relationship between the inhibitor level and FVIII level and also aPTT values [4,5,20,21].

### 4.4. When Do Inhibitors to FVIII Develop, or When Should Clinicians Suspect Inhibitor Development?

As noted above, FVIII inhibitors may develop in people with CHA at any time during treatment with FVIII replacement [4,5]. There is some debate about whether some forms of treatment (i.e., pdFVIII, rFVIII, EHL-FVIII) are more or less associated with inhibitor development, but all can be associated with inhibitor development, so clinicians should always be cognizant of the potential for the development of inhibitors whilst a patient is under therapy [4,5,20,21,22,32]. It is usual for patients to be monitored by assessing FVIII levels, either by one-stage or chromogenic FVIII assays [29,30,31]. Clinicians should keep a close eye on these test results, as a lower-than-expected FVIII level or a lower-than-expected therapeutic response (i.e., a bleed despite FVIII replacement) may be an indicator of FVIII inhibitor development. A further indicator is the patient in whom the severity of the bleeding appears to worsen, for example a mild hemophiliac who develops spontaneous bleeding.

For AHA, clues to the presence of a FVIII inhibitor are recent bleeding without identifiable cause or an unexpectedly raised aPTT, especially if an FVIII assay is performed and found to be low [3]. Bleeding at presentation of AHA is usually severe and spontaneous, with the most commonly affected sites being skin and deep tissue, including musculoskeletal and retroperitoneal bleeding. Hemarthrosis is much less common than for CHA. As noted above, AHA is associated with increasing age, and so a low FVIII found at a late-age presentation is unlikely to be due to CHA [3,32].

Figure 1 shows some sample relationships between FVIII level and aPTT from our locality. The general findings otherwise highlighted here (Figure 3, Figure 4 and Figure 5) complicate both clinical and laboratory test practice, since even a normal FVIII level may not always exclude the presence of an FVIII inhibitor (although it usually will), and a low level of FVIII does not necessarily reflect the presence of an FVIII inhibitor (since it may represent CHA or even von Willebrand disease [VWD]) [33].

## 5. Perspectives, Recommendations and Conclusions

Although potentially complex, the real-world data trends shown in the current perspective can also be used to enable some recommendations for laboratory practice. First, once an AHA patient has been identified, it is important to follow that patient until the FVIII inhibitor can no longer be identified [3,20,21]. Usually, the disappearance of the inhibitor will align to the normalization of aPTT and FVIII:C levels, but on occasion, inhibitors will continue to be identified even when these appear to be normal (Figure 4B).

Second, the in vitro method for assessing the FVIII sensitivity of an aPTT reagent appears to be a reasonable method (Figure 1). Although the ex vivo method (Figure 5A) provides greater inherent value, as this reflects data with real-world patients, this method takes considerable time to provide sufficient accumulation of data points and is really better as a confirmatory step to confirm sensitivity values estimated with the in vitro method [23,24,25]. Also, the in vitro method is the only feasible method for rarer coagulation factor deficiencies [24,25,26].

It is also worth noting that monitoring of inhibitor development may also depend on local guidance. For example, the authors practice in Australia and follow local guidance [22]. Some specific recommendations from this guidance for monitoring of inhibitors is provided in Table 1. These recommendations, in part, explain the large number of inhibitor assessments performed in our CHA population (Figure 5B) and why most of these assessments yield inhibitor negative findings.

Instead, for AHA patients, once an inhibitor is found, the laboratory is called upon to perform ongoing evaluation of the inhibitor until its disappearance. This also, in part, explains why most of the data points in Figure 4B show positive inhibitor findings and why inhibitor negative findings only occur when FVIII levels become normal. Also, due to the risk of inhibitor relapse, periodic monitoring for inhibitor redevelopment is performed following remission as part of a patient’s management (i.e., surveillance).

Of course, it is important to identify that this manuscript, as a perspective piece, reflects our personal perspectives based on local experience and applicable to us in Australia. Our experience may differ from those of others, who then may undertake alternate approaches. Thus, it is important to note the limitations of our perspective, and our experience, as identified in Figure 1, Figure 3, Figure 4 and Figure 5. We also note that the development of alternate therapies for hemophilia patients, both with and without inhibitors, may change the landscape of inhibitor assessment in the future.

Finally, in the age of bypass agents and FVIII mimetics, laboratories have to develop novel strategies to assess inhibitors in such treated patients [31]. For example, chromogenic assays using bovine reagents are required to monitor inhibitor development in emicizumab-treated patients. Clinicians should also be wary about certain additional treatments in emicizumab-treated patients, for example to avoid the use of factor eight inhibitor bypass activity (FEIBA), especially in high doses for CHA patients with inhibitors who have bleeding on emicizumab or require bypassing agents for surgery [34,35,36,37].

## Figures and Tables

**Table 1:** Some specific recommendations on inhibitor monitoring from the management of hemophilia in Australia guidelines *.

Point	Recommendation
6.2.10	In all cases, inhibitors render treatment with replacement factor concentrates difficult. Patients on clotting factor therapy should therefore be screened for development of inhibitors.
6.2.11	Confirmation of the presence of an inhibitor and quantification of the titer is performed in the laboratory, preferably using the Nijmegen-modified Bethesda assay.
6.2.12	For children, inhibitors should be screened once every 5 exposure days until 20 exposure days, then every 10 exposure days between 21 and 50 exposure days and at least two times a year until 150 exposure days
6.2.13	For adults with more than 150 exposure days, apart from a 6–12 monthly review, any failure to respond to adequate factor concentrate replacement therapy in a previously responsive patient is an indication to assess for an inhibitor.
6.2.14	Inhibitors should also be assessed in all patients who have been intensively treated for more than 5 days, within 4 weeks of the last infusion.
6.2.15	Inhibitors should also be assessed before surgery or if recovery assays are not as expected, and when clinical response to treatment of bleeding is suboptimal in the postoperative period.

* From reference [22].

## Data Availability

The raw data supporting the conclusions of this article will be made available by the authors upon reasonable request.
